# [^18^F]THK-5351 PET imaging in early-stage semantic variant primary progressive aphasia: a report of two cases and a literature review

**DOI:** 10.1186/s12883-018-1115-3

**Published:** 2018-08-08

**Authors:** Ryota Kobayashi, Hiroshi Hayashi, Shinobu Kawakatsu, Aiko Ishiki, Nobuyuki Okamura, Hiroyuki Arai, Koichi Otani

**Affiliations:** 1 Department of Psychiatry, Yamagata University School of Medicine, 2-2-2 Iidanishi, Yamagata City, Yamagata 990-9585 Japan; 2 Department of Neuropsychiatry, Aizu Medical Center, Fukushima Medical University, Fukushima, Japan; 3 Department of Geriatrics and Gerontology, Institute of Development, Aging and Cancer, Tohoku University, Sendai, Japan; 4 Cyclotron and Radioisotope Center, Tohoku University, Sendai, Japan; 5 Division of Pharmacology, Faculty of Medicine, Tohoku Medical and Pharmaceutical University, Sendai, Japan

**Keywords:** Semantic variant primary progressive aphasia, [^18^F]THK-5351 positron emission tomography imaging, Anomia, Prosopagnosia

## Abstract

**Background:**

Semantic variant primary progressive aphasia (svPPA) is a subtype of primary progressive aphasia characterized by two-way anomia and disturbance in word comprehension, with focal atrophy in the left temporal lobe. [^18^F]THK-5351 was originally developed to trace tau protein. However, it has recently been suggested that [^18^F]THK-5351 binds to monoamine oxidase B in astrocytes, which reflects gliosis. Herein, the authors present two cases involving patients with early-stage svPPA who underwent [^18^F]THK-5351 positron emission tomography (PET) imaging, and examined whether [^18^F]THK-5351 PET imaging is more sensitive to neurodegenerative lesions than conventional imaging modalities such as magnetic resonance imaging (MRI) and cerebral blood flow (CBF)-single photon emission computed tomography (SPECT).

**Case presentation:**

Two patients, 64- and 79-year-old men, without notable medical or family history, exhibited disturbances in word comprehension and mild anomia with fluent speech and spared repetition. In both cases, surface dyslexia was observed but prosopagnosia was absent. Although mild depression was detected in 1 of the 2 patients, no behavioral disorders were present in either case. In both cases, MRI revealed atrophy in the anterior and inferior portions of the left temporal lobe. Technetium-99-ethyl cysteinate dimer ([^99m^Tc]ECD) SPECT revealed hypoperfusion in the left temporal lobe. Alzheimer’s disease was ruled out by [^11^C]Pittsburgh Compound-B (PiB) PET scan. Both patients fulfilled the diagnostic criteria for svPPA. Because of mild language deficits and lack of right temporal atrophy, they were considered to be at an early stage of the disease. In both cases, [^18^F]THK-5351 retention was observed in bilateral temporal lobes, predominantly on the left side. Comparison of different imaging modalities suggested that [^18^F]THK-5351 was more sensitive in detecting neurodegenerative change in the right temporal lobe than MRI and [^99m^Tc]ECD SPECT.

**Conclusions:**

[^18^F]THK-5351 retention was clearly demonstrated at an early stage of svPPA. Results of the present study suggest that [^18^F]THK-5351 PET imaging may facilitate very early diagnosis of the disease.

**Electronic supplementary material:**

The online version of this article (10.1186/s12883-018-1115-3) contains supplementary material, which is available to authorized users.

## Background

Semantic variant primary progressive aphasia (svPPA) is a subtype of primary progressive aphasia that is characterized by anomia, disturbance in word comprehension, and other impairments, and presents with atrophy in the anterior area of the left temporal lobe [1]. Although this disorder is pathologically caused by frontotemporal lobar degeneration (FTLD) with inclusion bodies positive for transactivation responsive region DNA-binding protein of 43 Kda (TDP43) in most cases, it was reported that some cases could be attributed to a tauopathy, such as Pick’s disease or Alzheimer’s disease (AD) [2].

In tauopathies, such as AD or progressive supranuclear palsy, [^18^F]THK-5351 has been reported to be well retained [3–5]. However, it was recently indicated that [^18^F]THK-5351 may bind to monoamine oxidase B (MAO-B), which reflects astrogliosis [6].

Although few reported studies have used [^18^F]THK-5351 positron emission tomography (PET) imaging in svPPA patients, in whom tauopathy is supposedly rare, [^18^F]THK-5351 retention was observed in all studies [7–9]. Results of these studies supported the hypothesis that [^18^F]THK-5351 may detect astrogliosis rather than tau pathology. Previous PET studies involving patients in the advanced stages of svPPA have reported [^18^F]THK-5351 retention in the bilateral temporal lobes, accompanied by temporal lobe atrophy. Assuming that [^18^F]THK-5351 detects astrogliosis in svPPA, [^18^F]THK-5351 retention may appear earlier than brain atrophy or alterations in cerebral blood flow (CBF) [10]. In the present article, we present two cases of early-stage svPPA without right temporal atrophy. We examined whether [^18^F]THK-5351 PET imaging could detect neurodegenerative lesions with more sensitivity than conventional imaging techniques such as magnetic resonance imaging (MRI) and CBF-single photon emission computed tomography (SPECT).

## Case presentations

### Case 1

**Fig. 1:**
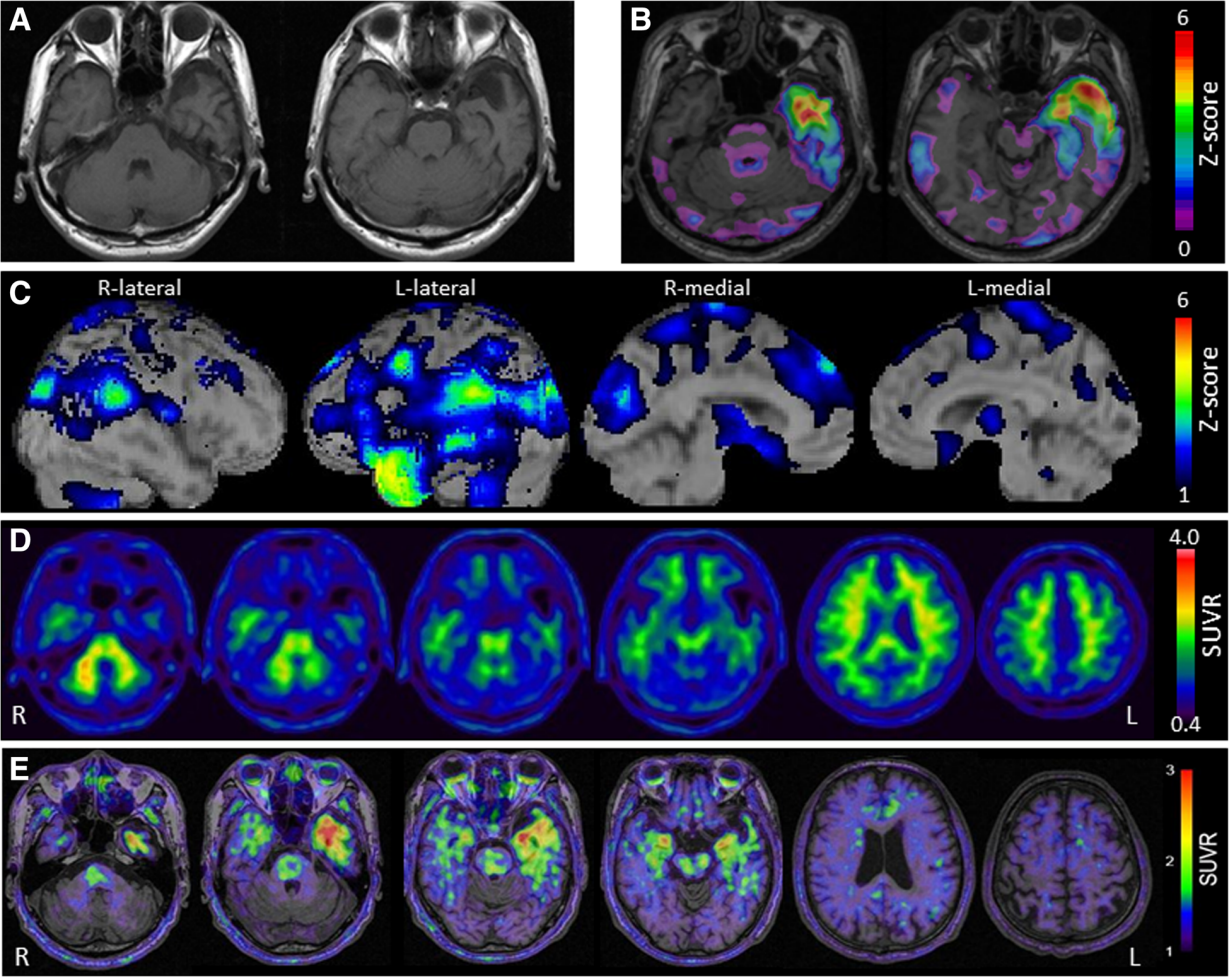
Neuroradiological findings in case 1. T1-weighted magnetic resonance imaging (**a**) revealing left-side dominant, marked focal atrophy in the anterior and inferior portions of the temporal lobes. Z-score maps (**b**) of gray matter loss were created by the comparison of individual gray matter images with the mean and standard deviation of gray matter images of 80 healthy controls after voxel normalization to global mean intensities, using voxel-based specific regional analysis system for AD (VSRAD) advance version. The color scale for the Z score is shown in the lower right part of the figure. Presented in color if the Z score is > 0. Brain SPECT eZIS analysis compared with 40 age-matched normal controls (**c**) revealed relative hypoperfusion mainly in the left anterior temporal areas. The color scale for the Z score is shown in the right part of the figure. Presented in color if the Z score is > 1. Amyloid positron emission tomography (PET) with [^11^C]PiB (**d**) did not reveal specific binding in the neocortical gray matter. The color scale for the standardized uptake value ratio (SUVR) is shown in the right part of the figure. [^18^F]THK-5351 PET (**e**) showed that [^18^F]THK-5351 retention was markedly elevated in the anterior and inferior portions of the left temporal lobe. The color scale for the SUVR is shown in the right part of the figure

The patient was a 64-year-old, right-handed man. He graduated from a junior high school at 15 years of age, and worked in a supermarket. There were no notable issues in either his medical or family history. At 60 years of age, he exhibited symptoms including the inability to name products and to comprehend in-store announcements. He visited the authors’ hospital for the first time in 2016, as his symptoms gradually interfered with his work. Proper conduct was maintained, and his spontaneous speech was fluent. However, word-finding difficulty was detected. According to the Neuropsychiatric Inventory (NPI), he had mild depression, possibly caused by his inability to understand the meaning of words, but did not exhibit any behavioral disorders or other findings. Neuropsychological assessment revealed mild anomia, disturbance in word comprehension, and spared repetition (Table 1). In the object-naming subtest of the Western Aphasia Battery (WAB), some tasks revealed anomia, which was not improved after the cues of the initial sound of the words. In the vocabulary subtest of the Wechsler Adult Intelligence Scale-III (WAIS-III), he was unable to provide the definitions of low-frequency words. In the Kanji (Japanese morphogram) reading task, surface dyslexia was detected. He was unable to understand the meanings of idioms and proverbs. Because auditory word recognition was preserved in the Standard Language Test of Aphasia (SLTA), semantic aphasia was considered to be mild. In the face-recognition subtests of the Visual Perception Test for Agnosia (VPTA), prosopagnosia was not detected. Additionally, there was no impairment in recognizing the faces of family members or acquaintances in daily life. No abnormalities were detected in the neurological examination. MRI revealed atrophy in the anterior and inferior portions of the left temporal lobe. Technetium-99-ethyl cysteinate dimer ([^99m^Tc]ECD) SPECT revealed hypoperfusion in the anterior area of the left temporal lobe. The visual assessment of [^11^C]Pittsburgh Compound-B (PiB) PET scans, based on the Japanese Alzheimer’s Disease Neuroimaging Initiative (J-ADNI) protocol [11], yielded negative results. His apolipoprotein E phenotype was E3/5 or E3/7. He was diagnosed with svPPA based on the diagnostic criteria developed by Gorno-Tempini et al. [1]. His naming impairment and word comprehension deficits were mild. Moreover, he presented no atrophy in the right temporal lobe. Accordingly, this patient was considered to be in an early stage of the disease. [^18^F]THK-5351 PET imaging revealed significant [^18^F]THK-5351 retention in the bilateral temporal lobes, predominantly on the left side (Fig. 1 and Additional file 1: Figure S1). Structural MRI revealed brain atrophy in the left anterior temporal pole. [^99m^Tc]ECD SPECT scan also revealed unilateral hypoperfusion in the left anterior temporal pole. For the comparison of different imaging modalities, Z-score maps of [^18^F]THK-5351 PET were created by the comparison of individual PET images with the mean and standard deviation of 20 normal controls, using PMOD software (PMOD Technologies, Zürich, Switzerland). Z-score maps of CBF-SPECT and voxel-based morphometry (VBM)-MRI were also created using easy Z-score imaging system (eZIS) software (Fujifilm RI Pharma., Tokyo, Japan) and voxel-based specific regional analysis system for AD (VSRAD) software (Eisai, Tokyo, Japan) [12, 13]. In the right temporal lobe, the Z-score of [^18^F]THK-5351 PET (Z = 3.70) was greater than that of CBF-SPECT (Z < 1.0) and VBM-MRI (Z < 1.0).

**Table 1:** Neuropsychological assessment

Scores of cognitive tests	Case 1	Case 2	Non-aphasic controls
Mini Mental State Examination (/30)	29	28	
Frontal Assessment Battery (/18)	18	18	
Western Aphasia Battery Object naming (/60)	45	46	59.2 ± 2.4
Standard Language Test of Aphasia			
Auditory word recognition (/10)	10	10	10.0 ± 0.2
Naming (/20)	16	15	19.6 ± 0.8
Word repetition (/10)	10	10	10.0 ± 0.1
Verbal fluency (/15)(animal/min)	10	6	12.6 ± 4.5
Wechsler Adult Intelligence Scale-III			
Vocabulary (/64)	19	20	
Digit span Forward (/16)	8	10	
Rey-Osterrieth Complex Figure Test			
Copying (/36)	36	35	
Delayed recall (/36)	18	10	
Visual Perception Test for Agnosia			
Famous face naming (/16)	14	16	
Famous face pointing (/16)	0	2	
Neuropsychiatric Inventory (/120)	1	0	

The Visual Perception Test for Agnosia is a comprehensive test battery for visual perception standardized for Japanese, in which 0 denotes that one completely recognizes the faces of eight famous people, and 16 denotes that one cannot recognize any of the faces

### Case 2

**Fig. 2:**
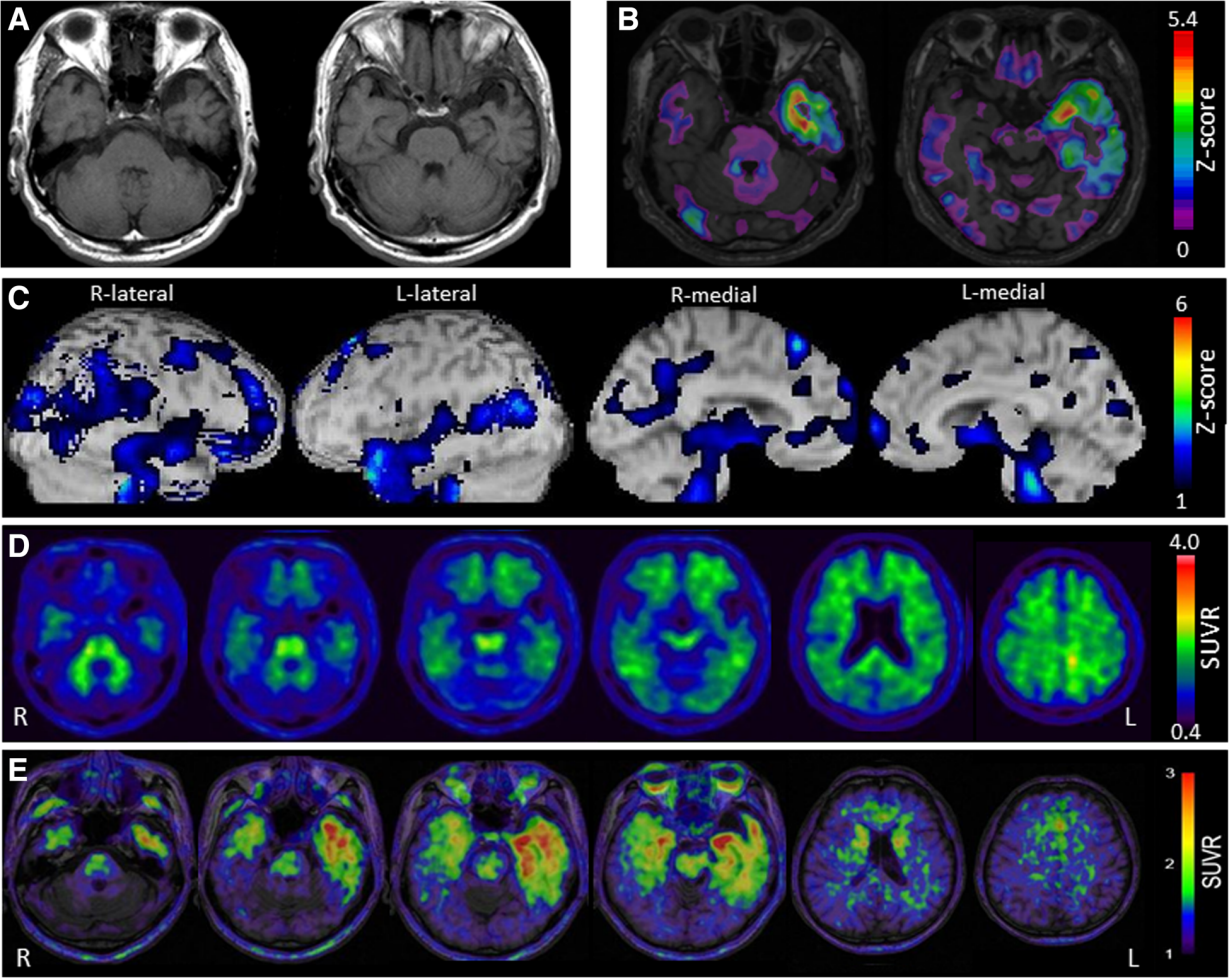
Neuroradiological findings in case 2. T1-weighted magnetic resonance imaging (**a**) shows left-side dominant, marked focal atrophy in the anterior and inferior portions of the temporal lobes. Z-score maps (**b**) of gray matter loss were created by the comparison of individual gray matter images with the mean and standard deviation of gray matter images of 80 healthy controls after voxel normalization to global mean intensities, using voxel-based specific regional analysis system for AD (VSRAD) advance version. The color scale for the Z score is shown in the lower right part of the figure. Presented in color if the Z score is > 0. Brain SPECT eZIS analysis compared with 40 age-matched normal controls (**c**) revealed relative hypoperfusion mainly in the left anterior temporal areas. The color scale for the Z score is shown in the right of the figure. Presented in color if the Z score is > 1. [^11^C] PiB-PET (**d**) revealed extremely mild accumulation confined to the left parietal lobe; however, it was atypical and, therefore, a diagnosis of Alzheimer’s disease was not considered. The color scale for the standardized uptake value ratio (SUVR) is shown in the right part of the figure. [^18^F]THK-5351 PET (**e**) revealed that [^18^F]THK-5351 retention was markedly elevated in the anterior and inferior portions of the left temporal lobe. The color scale for the SUVR is shown in the right part of the figure

The patient was a 79-year-old, right-handed man with 16 years of education. There were no notable issues in either his medical or family history. When he was 77 years of age, his wife noticed that he became unable to name objects used in daily life. During the next two years, his language symptoms had gradually worsened, and he visited the authors’ hospital in 2017. Although his spontaneous speech was fluent and proper conduct was maintained, word-finding difficulty was evident, and his episodic memory in daily life was preserved. According to the NPI, there was no behavioral disorder. Neuropsychological assessment revealed mild anomia, disturbance in word comprehension, and spared repetition (Table 1). In the object-naming subtest of the WAB, some tasks revealed anomia, which was not improved after the cues of the initial sound of the words. In the vocabulary subtest of the WAIS-III, he was unable to provide the definitions of low-frequency words. In the Kanji (Japanese morphogram) reading task, surface dyslexia was detected. He was unable to understand the meanings of idioms and proverbs. Because auditory word recognition was preserved in SLTA, semantic aphasia was considered to be mild. In the face-recognition subtests of the VPTA, prosopagnosia was not detected. Additionally, there was no impairment in recognizing the faces of family members and acquaintances in daily life. No abnormalities were detected on the neurological examination. MRI revealed atrophy in the anterior and inferior portions of the left temporal lobe. [^99m^Tc]ECD SPECT scan revealed hypoperfusion in the left temporal lobe. [^11^C]PiB-PET scan was considered to be negative according to the J-ADNI protocol [11], although very mild and focal [^11^C]PiB retention was observed only in the parietal lobe. His apolipoprotein E phenotype was E3/3. He was diagnosed with svPPA based on the diagnostic criteria developed by Gorno-Tempini et al. [1]. He was considered to be in an early stage of svPPA because of his mild anomia and word comprehension disturbance, and no remarkable atrophy in the right temporal lobe. In [^18^F]THK-5351 PET scan, [^18^F]THK-5351 retention was observed in the bilateral temporal lobes, predominantly in the left side (Fig. 2 and Additional file 1: Figure S1). In an analysis similar to case 1, Z-score in the right temporal pole was greater in [^18^F]THK-5351 PET (Z = 1.27) than in CBF-SPECT (Z < 1.0) and VBM-MRI (Z < 1.0).

## Discussion and conclusions

In the present study, patients with svPPA exhibited [^18^F]THK-5351 retention in the bilateral temporal lobes, predominantly on the left side. This finding is consistent with previous observations in a total of 7 cases (Table 2). Because the 2 cases in the present study exhibited no remarkable amyloid deposition on PiB-PET scan, it is unlikely that paired helical filament (PHF)-tau was highly accumulated in the neocortex of these individuals. Therefore, [^18^F]THK-5351 retention in the temporal lobe of these patients may be explained by the binding to non-PHF tau or TDP43. Takaya et al. interpreted the results of negativity for PiB and positivity for [^18^F]THK-5351, which were also found in the present study, as FTLD with tau-positive inclusion bodies (FTLD-tau) [7]. However, svPPA is frequently associated with TDP43 proteinopathy and weakly associated with tauopathies such as Pick’s disease [14]. An in vitro study [15] reported low binding affinity of [^18^F]THK-5351 to 3R-tau and TDP43; accordingly, we should consider the possibility of [^18^F]THK-5351 binding to the other target. Ng et al. performed [^18^F]THK-5351 imaging in patients who were administered MAO-B inhibitors and observed decreases in the uptake of [^18^F]THK-5351, suggesting the binding of THK-5351 to MAO-B [16]. Harada et al. reported that [^18^F]THK-5351 retention in autopsy-confirmed AD cases correlated with the density of tau aggregates and MAO-B, and that there was a significant association among tau aggregates, MAO-B, and glial fibrillary acidic protein [6]. Based on these results, they proposed that [^18^F]THK-5351 PET imaging could be used for monitoring the neuroinflammatory processes, although [^18^F]THK-5351 has limited utility as a biomarker for tau pathology in AD [6]. It was also reported that [^18^F]THK-5351 retention correlates with decreased glucose metabolism on [^18^F]fluorodeoxyglucose PET and atrophy on MRI in svPPA [8]. In light of these previous findings, we believe that [^18^F]THK-5351 retention in our svPPA cases reflected MAO-B positive astrogliosis.

**Table 2:** Clinical findings of patients with svPPA who underwent [^18^F]THK-5351 PET imaging

Reference	Sex/age (years)	Duration (years)	MMSE (/30)	Clinical presentation	Atrophy- predominant	MRI findings	THK-5351 retention
Takaya M et al., 2017 [7]	F/69	5	12	Comprehension difficulty, surface dyslexia	L > R	Bilateral anterior temporal lobe atrophy	Bilateral anterior temporal regions
Lee H et al., 2017 [8]	F/79	4	8 (average of 5 cases)	Comprehension difficulty, compulsive behavior	L > R	Bilateral anterior temporal lobe atrophy	Bilateral anterolateral temporal regions
	F/76	3	8 (average of 5 cases)	Anomia, comprehension difficulty, inappropriate social behavior	R > L	Bilateral anterior temporal lobe atrophy	Bilateral anterolateral temporal regions
	M/50	10	8 (average of 5 cases)	Anomia, comprehension difficulty, prosopagnosia, behavioral changes	L > R	Bilateral anterior temporal lobe atrophy	Bilateral anterolateral temporal regions
	F/60	1	8 (average of 5 cases)	Comprehension difficulty, prosopagnosia, behavioral changes	L > R	Bilateral anterior temporal lobe atrophy	Bilateral anterolateral temporal regions
	M/66	2	8 (average of 5 cases)	Anomia, comprehension difficulty	L > R	Bilateral anterior temporal lobe atrophy	Bilateral anterolateral temporal regions
Jang YK et al., 2017 [9]	M/68	NA	20	Decreased understanding ability, confusion about names of objects	R > L	Bilateral anterior temporal lobe atrophy	Bilateral anterior and inferior temporal regions

*F* female, *L* left, *M* male, *MRI* magnetic resonance imaging, *NA* not available, *R* right*, svPPA* semantic variant primary progressive aphasia, THK-5351: [^18^F]THK-5351

Advanced svPPA presents with atrophy in the temporal lobe, and not only on the left side, but also on the right side, causing prosopagnosia resulting from right temporal lobe damage, and stereotype disorders and other behaviors [17]. In previous studies, five patients with advanced svPPA exhibited left-predominant temporal lobe atrophy and also exhibited marked atrophy in the right temporal lobe. Moreover, in two patients with right-predominant temporal lobe atrophy, it was shown that left temporal lobe atrophy was as remarkable as it was on the right side. In all patients, [^18^F]THK-5351 retention was more pronounced on the predominant side of atrophy, while it was also observed in the contralateral temporal lobe [7–9]. The present study was the first to report [^18^F]THK-5351 PET imaging performed in patients with early-stage svPPA without right temporal lobe atrophy, accompanied by mild anomia and disturbance in word comprehension, but not by prosopagnosia or behavioral disorder. Interestingly, [^18^F]THK-5351 retention was observed not only in the left temporal lobe but also in the anterior area of the right temporal lobe without atrophy and hypoperfusion. The results of Z-score analysis suggested that [^18^F]THK-5351 PET could be highly sensitive to mild neurodegenerative lesions that cannot be detected using VBM-MRI or CBF-SPECT.

One limitation of the present study was that the interval between MRI or SPECT scans and [^18^F]THK-5351 PET imaging was 10 months in case 1 and 7 months in case 2. Thus, when [^18^F]THK-5351 PET imaging was performed, the abnormality in the right temporal lobe may have been detectable by MRI or SPECT at the time of [^18^F]THK-5351 PET scan. However, because there was no clinical finding suggestive of right temporal lobe impairment at that time, it is unlikely that advanced neurodegeneration existed in the right temporal lobe.

The present study demonstrated [^18^F]THK-5351 retention in the right temporal lobe in the absence of symptoms associated with right temporal lobe impairment, suggesting that [^18^F]THK-5351 retention in the temporal lobe may be detectable in the very early stages of svPPA before clinical symptoms appear. In other words, the results of the present study indicated that [^18^F]THK-5351 PET imaging may be useful for attaining an very early diagnosis of svPPA.

## Additional file

Additional file 1: **Figure S1.** THK-5351 PET images in a healthy control (81-year-old female), Alzheimer’s disease (80-year-old male), and the current cases. All participants signed a written consent. (TIF 4617 kb)

## Abbreviations

[99 m]Tc-ECD: Technetium-99-ethyl cysteinate dimer
AD: Alzheimer’s disease
eZIS: easy Z-score imaging system
FTLD: Frontotemporal lobar degeneration
J-ADNI: Japanese Alzheimer’s Disease Neuroimaging Initiative
MAO-B: monoamine oxidase B
MRI: Magnetic resonance imaging
NPI: Neuropsychiatric Inventory
PET: Position emission tomography
PiB: Pittsburgh Compound-B
SLTA: Standard Language Test of Aphasia
SPECT: Single photon emission computed tomography
svPPA: semantic variant primary progressive aphasia
TDP43: Transactivation responsive region DNA-binding protein of 43 Kda
VPTA: Visual Perception Test for Agnosia
VSRAD: Voxel-Based Specific Regional Analysis System for Alzheimer’s Disease
WAB: Western Aphasia Battery
WAIS-III: Wechsler Adult Intelligence Scale-III
