# Supplementary figures and images for: [18F]THK-5351 PET imaging in early-stage semantic variant primary progressive aphasia: a report of two cases and a literature review
Source: BMC Neurol. 2018 Aug 8;18:109. doi: 10.1186/s12883-018-1115-3 (PMC6205153; doi:10.1186/s12883-018-1115-3)

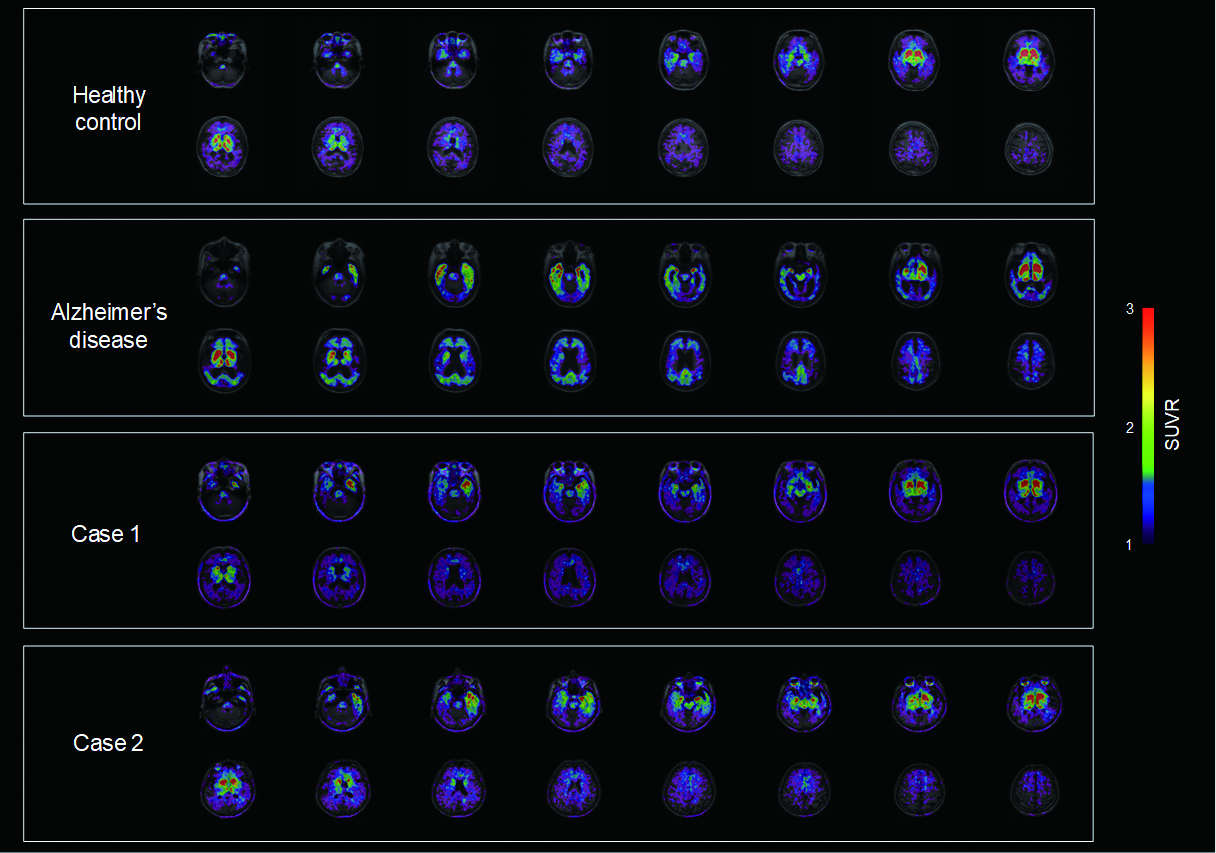

Supplement: Supplementary file 1 — Figure S1. THK-5351 PET images in a healthy control (81-year-old female), Alzheimer’s disease (80-year-old male), and the current cases. All participants signed a written consent. (TIF 4617 kb) [file 12883_2018_1115_MOESM1_ESM.tif]
